# Utilization of Alanine Dosimetry for 10 MV Photon Beam Dose Evaluation

**DOI:** 10.3390/molecules31060971

**Published:** 2026-03-13

**Authors:** HyoJin Kim, Jeung Kee Kim, Jieun Lee, Hee Jin Jang, Yong-Uk Kye, Jeong-Hwa Baek, Wol Soon Jo, Doyoung Jung, Yeong-Rok Kang

**Affiliations:** Dongnam Institute of Radiological & Medical Sciences, Jwadong-gil 40, Jangan-eup, Gijang-gun, Busan 46033, Republic of Korea; kimhyojin@dirams.re.kr (H.K.); jkkim@dirams.re.kr (J.K.K.); lje5906@dirams.re.kr (J.L.); heejin00@dirams.re.kr (H.J.J.); kyu0610@dirams.re.kr (Y.-U.K.); jihan918@dirams.re.kr (J.-H.B.); sailorjo@dirams.re.kr (W.S.J.); jdy0415@dirams.re.kr (D.J.)

**Keywords:** electron paramagnetic resonance (EPR)/alanine dosimetry, radiation therapy, quality control, LINAC, dose-response curve

## Abstract

Radiation therapy is a crucial treatment method that delivers a high dose of radiation to localized areas. Therefore, ensuring the accuracy of the radiation dose from the radiation generator is essential. Alanine dosimetry offers the advantage of being equivalent to water and tissue and is thus a valuable tool for estimating radiation doses in the human body. In this study, we aimed to assess the feasibility of utilizing electron paramagnetic resonance (EPR)/alanine dosimetry for quality control of medical linear accelerators (LINACs). The EPR signal and slope of the dose-response curve were compared with the number of alanine dosimeters per dose using a 10 MV linear accelerator, and the measurement uncertainty was evaluated. The signal increased by approximately 0.03 per 1 Gy per count, in conjunction with the slope of the dose-response curve. The measurement uncertainty, estimated based on the synthesis of eight numerical factors of the uncertainty propagation law, was approximately 2.49–4.19% (k = 1). The findings of this study suggest that the EPR/alanine dosimetry system can be reliably applied for quality control of 10 MV photon beams under the investigated experimental conditions.

## 1. Introduction

Radiation therapy has significantly improved in effectiveness owing to technological advancements, such as intensity-modulated radiation therapy, volumetric-modulated arc therapy, image-guided radiation therapy, and respiratory-gated radiation therapy. These advancements have enhanced the therapeutic efficacy of the procedure, leading to its increased utilization in various cancer treatment modalities [1,2].

The goal of radiation therapy is to treat cancer by delivering a precise and high dose of radiation to the affected area while minimizing exposure to surrounding healthy tissue. Additionally, in the realm of diagnostic radiology, ensuring the accuracy and reliability of radiation dose delivery is crucial, as high doses are directly administered to patients. Therefore, in clinical practice, quality control measures are routinely implemented on medical linear accelerators following established procedures [3,4,5,6].

One method of assessing radiation dose is by utilizing an alanine dosimeter, which operates based on the principle of EPR. When exposed to radiation, the alanine dosimeter generates free radicals in proportion to the dose received, which can be detected by an EPR spectrometer for dose evaluation. Alanine dosimeters offer the advantage of being water and tissue equivalent, making them ideal for assessing dose exposure in humans [7,8,9]. Therefore, various studies have utilized alanine dosimeters to evaluate dose exposure in humans [10,11].

Alanine dosimetry has been widely investigated and established as a reliable secondary standard and transfer dosimetry system in radiotherapy. International organizations such as the International Atomic Energy Agency (IAEA) have evaluated its applicability for reference and audit dosimetry, and previous studies have thoroughly assessed its measurement uncertainty and clinical feasibility. Building upon this established foundation, the present study focuses on evaluating the feasibility of EPR/alanine dosimetry for quality control of 10 MV photon beams under specific experimental conditions. Additionally, studies on dose assessment of the alanine-free dosimeter at clinical dose levels using a 6 MV X-ray beam from a LINAC have been performed, as well as studies comparing the alanine dosimeter response to the LINAC and gamma knife [12,13]. Previous studies have primarily focused on specific irradiation conditions and on mass normalization procedures to compensate for pellet-to-pellet variations. While the proportional relationship between EPR signal intensity and alanine mass is well recognized, comparatively limited attention has been given to the deliberate use of multiple stacked alanine dosimeters as a practical sensitivity enhancement strategy under clinical megavoltage photon beam conditions. In particular, a systematic evaluation of how increasing the total effective mass influences the dose-response slope, measurement uncertainty, and practical applicability in quality control scenarios remains limited.

Therefore, this study evaluates the applicability of alanine dosimeters for quality control of medical linear accelerators under the investigated experimental conditions. We also analyzed the measurement results based on the number of alanine dosimeters used to recommend the optimal alanine mass for accurate measurements.

Although alanine dosimetry has been well established in radiotherapy applications, limited attention has been given to the quantitative evaluation of the effect of simultaneously increasing the total alanine dosimeter mass during EPR measurements. In particular, the relationship between mass-dependent signal sensitivity and measurement uncertainty under clinical 10 MV photon beam conditions has not been systematically analyzed. Therefore, this study aims to investigate the influence of total dosimeter mass on EPR signal response, dose-response characteristics, and associated measurement uncertainties, with the objective of providing practical considerations for quality control applications.

## 2. Results

In this study, we utilized an EPR/alanine dosimetry system to control the quality of medical linear accelerators.

### 2.1. Comparison of EPR Signals Based on Dose and Number

**Figure 1:**
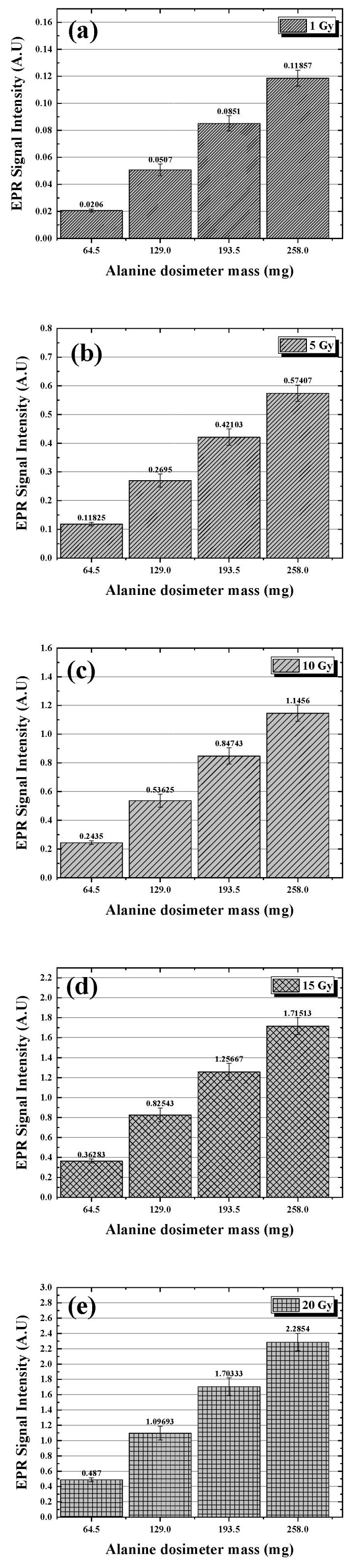
EPR signal intensity as a function of the total mass of alanine dosimeters. Each dosimeter has an average mass of 64.5 mg. (**a**) 1 Gy, (**b**) 5 Gy, (**c**) 10 Gy, (**d**) 15 Gy, and (**e**) 20 Gy.

The measured EPR signal and uncertainty based on the number of alanine dosimeters at a dose of 1 Gy are shown in Figure 1a. The signals based on number gradually increased to 0.02 ± 0.00, 0.05 ± 0.00, 0.09 ± 0.01, and 0.12 ± 0.01, with an increment of approximately 0.03. The measured EPR signal and uncertainty based on the number of alanine dosimeters at a dose of 5 Gy are shown in Figure 1b. The signals based on number were 0.12 ± 0.01, 0.27 ± 0.02, 0.42 ± 0.03, and 0.57 ± 0.03, with an increment of approximately 0.15. The measured EPR signal and the uncertainty based on the number of alanine dosimeters at a dose of 10 Gy are shown in Figure 1c. The signals based on number were 0.24 ± 0.01, 0.54 ± 0.04, 0.85 ± 0.06, and 1.15 ± 0.06, with an increment of approximately 0.30. The measured EPR signal and uncertainty based on the number of alanine dosimeters at a dose of 15 Gy are shown in Figure 1d. The signals based on number were 0.36 ± 0.02, 0.83 ± 0.07, 1.26 ± 0.08, and 1.72 ± 0.09, with an increment of approximately 0.45. The measured EPR signal and uncertainty based on the number of alanine dosimeters at a dose of 20 Gy are shown in Figure 1e. The signals based on number were 0.49 ± 0.03, 1.10 ± 0.09, 1.70 ± 0.11, and 2.29 ± 0.11, with an increment of approximately 0.60.

The EPR signal based on the number of alanine dosimeters demonstrated a proportional increase in the EPR signal with increasing number at all doses, with increases of 0.03, 0.15, 0.30, 0.45, and 0.60 at each dose, all of which were consistent with a 0.03 increase in signal per Gy.

### 2.2. Comparison of Dose-Response Curves Based on Alanine Dosimeter Mass

**Figure 2:**
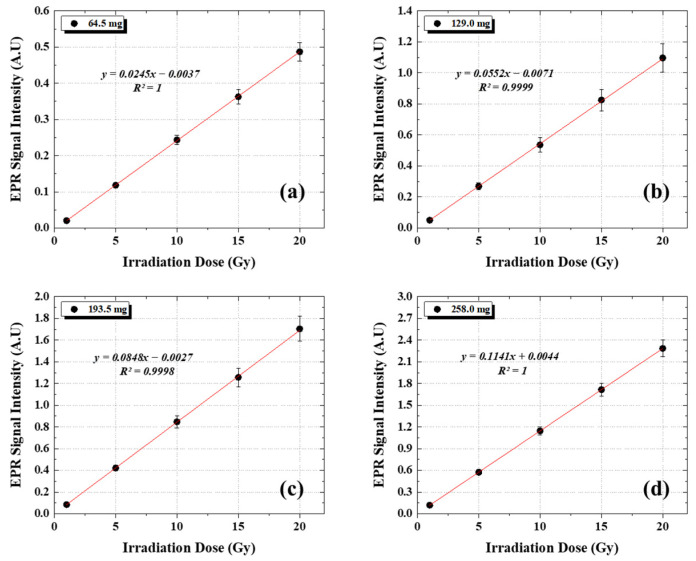
Mass-dependent dose-response curve of alanine dosimeters at 10 MV LINAC: (**a**) 64.5 mg, (**b**) 129.0 mg, (**c**) 193.5 mg, and (**d**) 258.0 mg alanine dosimeters.

Figure 2 compares the dose-response curves of alanine dosimeters with different masses (64.5–258.0 mg) irradiated using a 10 MV LINAC. For all investigated masses, the EPR signal intensity increased linearly with absorbed dose over the range of 0–20 Gy, and linear regression analysis yielded coefficients of determination (R^2^) greater than 0.9998, indicating excellent linearity.

Specifically, the slopes were 0.0245 a.u. Gy^−1^ (R^2^ = 1.0000) for the 64.5 mg dosimeter (Figure 2a), 0.0552 a.u. Gy^−1^ (R^2^ = 0.9999) for the 129.0 mg dosimeter (Figure 2b), 0.0848 a.u. Gy^−1^ (R^2^ = 0.9998) for the 193.5 mg dosimeter (Figure 2c), and 0.1141 a.u. Gy^−1^ (R^2^ = 1.0000) for the 258.0 mg dosimeter (Figure 2d).

Although high linearity was maintained for all dosimeter masses (R^2^ ≥ 0.9998), the slope increased monotonically with increasing dosimeter mass. Overall, the sensitivity increased from 0.0245 to 0.1141 a.u. Gy^−1^ as the mass increased from 64.5 to 258.0 mg, corresponding to an absolute increase of 0.0896 a.u. Gy^−1^ and an approximately 4.7-fold enhancement.

**Figure 3:**
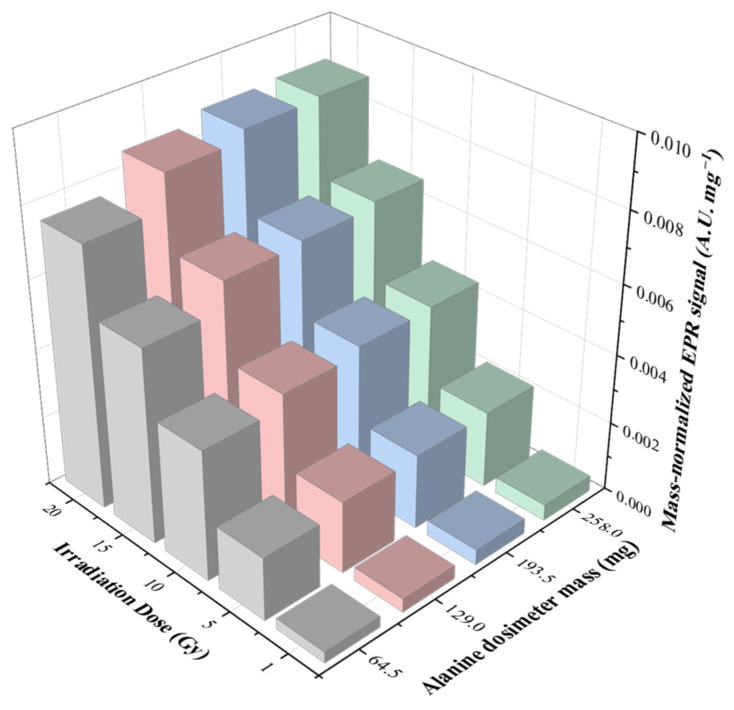
Mass-normalized EPR signal intensity as a function of irradiation dose and total alanine dosimeter mass.

Figure 3 presents the mass-normalized EPR response (Signal/Mass) as a function of the number of alanine pellets. While the total EPR signal exhibited a linear correlation with mass, the sensitivity per unit milligram showed a gradual increase as the number of stacked pellets increased. For example, at 10 Gy, the normalized signal increased from 0.00378 to 0.00444 a.u. mg^−1^, corresponding to an enhancement of approximately 18% between the single- and four-pellet configurations. This behavior is attributed to resonator filling factor effects and improved microwave coupling efficiency associated with the increased effective sample volume, rather than to changes in intrinsic alanine sensitivity. Importantly, the dose-response curves remained highly linear (R^2^ ≥ 0.9998) across all configurations, indicating the absence of significant saturation or intrinsic non-linear material effects within the investigated mass range.

### 2.3. Measurement Uncertainty of Dose-Response Curves

To ensure reliability, the measurement uncertainty of the dose-response curves was assessed. The uncertainty of the repeated measurements of the EPR signal intensity was evaluated based on 10 Gy, the middle value of the dose-response curve of the alanine dosimeter.

The measurement uncertainties of repeatability from repeated measurements based on the irradiated mass of the alanine dosimeters are listed in Table 1. The uncertainties were determined to be 0.2418 ± 0.0014, 0.5474 ± 0.0046, 0.8574 ± 0.0050, and 1.1402 ± 0.0030, based on the number. When expressed as relative uncertainties, these values were calculated to be 0.58%, 0.84%, 0.595, and 0.27%, respectively.

The uncertainty of the scale, its resolution, and the uncertainty of mass determination with repeated mass measurements based on the number of irradiated alanine dosimeters are listed in Table 2. Utilizing the same scale, the applied uncertainty and resolution were 0.3 and 0.06, respectively. The results of the repeated measurements based on number were 64.8 ± 0.00, 129.6 ± 0.00, 194.3 ± 0.04, and 259.0 ± 0.00. When synthesized and calculated as relative uncertainties, they were evaluated to be 0.47%, 0.24%, 0.16%, and 0.12%, respectively.

The uncertainty of the calibration curve, derived from the deviations between the measured EPR signal and the values calculated from the dose-response interpolation function, is summarized in Table 3.

The interpolation function was constructed using the measured EPR signal values, and the residuals (i.e., the differences between measured and interpolated values) were evaluated as a source of uncertainty. The standard deviation of the interpolation function, *u*(SDF), was calculated according to Equation (1):

(1) $$u\left(SDF\right)=\sqrt{\frac{\sum {\left[100\cdot \frac{\dot{K_{i}}-\dot{K_{f}}}{\dot{K_{f}}}\right]}^{2}}{n-m}}$$

where $\dot{K_{i}}$ represents the measured EPR signal, $\dot{K_{f}}$ denotes the interpolated value obtained from the dose-response curve, *n* is the number of data points, and *m* is the number of fitting parameters in the interpolation function.

Calculated as relative uncertainties through error, they were evaluated to be 0.68%, 3.27%, 2.22%, and 0.10%. The overall uncertainty was synthesized from the eight factors using the law of uncertainty propagation, and the relative scaling uncertainty was evaluated to be approximately 5.33%, 8.38%, 6.75%, and 4.97% (k = 2), depending on the number of factors.

## 3. Discussion

In this study, an EPR/alanine dosimetry system was utilized for the quality control of medical linear accelerators. The EPR signals and dose-response curves were compared based on the dose and number of alanine dosimeters using the 10-MV energy of the LINAC. Subsequently, the uncertainty of the dose-response curve was assessed.

Analysis of the EPR signal based on the number of alanine dosimeters revealed a proportional increase with a constant size of approximately 0.03 per Gy. This increase in the EPR signal was attributed to an increase in the number of free radicals detected by the EPR spectrometer. Anton compared EPR signals after correcting for differences in alanine mass and indicated a proportional relationship between mass and EPR signals, consistent with the experimental results of this study [14].

The dose-response curves, based on the number of alanine dosimeters, were evaluated with a coefficient of determination greater than 0.9998, validating their linear regression equations. Notably, the slope increased proportionally with the number. This phenomenon was attributed to the steady increase in the ratio of radiation dose to EPR signal with an increasing number of dosimeters, resulting in a steeper slope of the dose-response curve. This finding aligns with those of a study conducted by the National Institute of Standards and Technology in the U.S., which demonstrates that the sensitivity of EPR/alanine dosimetry systems is directly proportional to the number of dosimeters and dose [15]. This agreement between the results confirms that sensitivity and dose precision increase when alanine dosimeters are simultaneously utilized.

The assessment of measurement uncertainty, aimed at determining the reliability of the dose-response curve, yielded a range of approximately 2.49–4.19% (k = 1). The primary contributor to increased uncertainty was identified as the reference irradiation uncertainty, which encompassed all uncertainty factors associated with the LINAC, including dose and voltage. Notably, Hjørringgaard et al. reported an uncertainty of 4.6% (k = 1) in alanine dosimetry using irradiation devices [16]. A summary of the individual uncertainty components and the combined uncertainty is presented in Table 4. Therefore, the dose-response curves generated in this study were deemed reliable. A decreasing trend in relative uncertainty was observed with increasing absorbed dose. This behavior can be attributed to the proportional increase in EPR signal intensity with dose, resulting in an improved signal-to-noise ratio. As the absolute measurement uncertainty remains relatively constant, the relative uncertainty decreases at higher dose levels. This tendency has been consistently reported in previous alanine dosimetry studies and reflects the intrinsic statistical characteristics of EPR signal detection [14,15,16].

The present study was performed using a 10 MV photon beam, which is routinely employed as a reference beam for quality control and calibration at our institution. The choice of 10 MV was based on standardized and reproducible dosimetric conditions rather than on any specific energy-dependent characteristics of alanine.

Alanine dosimeters are well known to exhibit minimal energy dependence within the megavoltage photon range. According to established literature and ASTM 51607, the response variation between 6 and 10 MV photon beams is typically within a few percent, which is smaller than the combined measurement uncertainty evaluated in this study. Therefore, although absolute calibration factors may vary slightly with beam quality, the observed mass-dependent trends in dose-response slope and uncertainty behavior are not expected to differ significantly for 6 MV beams.

Although the minimum investigated dose in this study was 1 Gy in accordance with ASTM 51607 recommendations, it is noteworthy that the linear increase in EPR signal with both dose and dosimeter mass suggests improved signal-to-noise characteristics when multiple alanine dosimeters are used. This indicates that the practical detection limit may be reduced in multi-pellet configurations. A systematic determination of the Limit of Detection (LOD) and Limit of Quantification (LOQ) in the sub-Gy region will be addressed in future investigations to expand applicability to out-of-field and shielding dose assessments.

The enhancement in sensitivity achieved by increasing the number of dosimeters, however, involves a quantifiable trade-off with spatial resolution. The alanine dosimeters used in this study (5 mm in diameter and 3 mm in height) have an effective measurement volume (V_eff_) of approximately 58.9 mm^3^ per pellet. When four pellets are stacked to increase sensitivity, the total V_eff_ increases to approximately 235.6 mm^3^, resulting in an effective measurement length of approximately 12 mm along the stacking axis.

In regions with steep dose gradients, the measured signal represents a spatial average over the entire sensitive volume. For a typical 10 MV photon beam, the 80–20% penumbra width is generally reported to be approximately 3–5 mm. Therefore, a 12 mm stacked configuration integrates dose over a distance exceeding the characteristic gradient width, which may lead to partial smoothing of the measured dose profile. Accordingly, while multi-pellet configurations are advantageous for improving signal sensitivity and reducing relative uncertainty in uniform irradiation fields, configurations with fewer pellets may be more appropriate for applications requiring high spatial resolution, such as penumbra characterization or detailed dose distribution analysis. Optimization of the pellet number should therefore be based on the specific quality assurance objective, balancing sensitivity enhancement with spatial resolution and instrumental constraints

In the context of quality control for therapeutic radiation generators, the EPR/alanine dosimetry system is not intended to replace conventional QA tools used for beam profile, flatness, symmetry, or penumbra measurements. Rather, it serves as a supplementary and independent dosimetric verification method for output constancy and reference dose validation under uniform irradiation conditions. Owing to the increased effective measurement volume in multi-pellet configurations, the system is more suitable for absolute or relative dose verification than for high spatial resolution dose distribution assessment.

## 4. Materials and Methods

**Figure 4:**
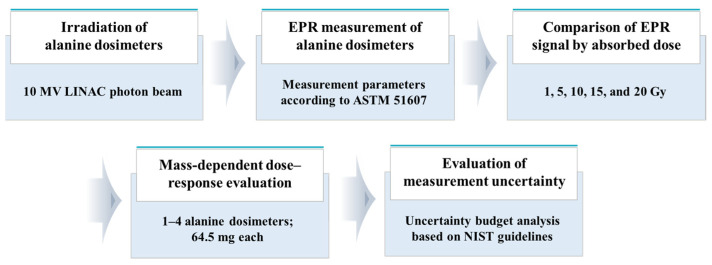
Flowchart of the experimental method.

In this study, we analyzed the EPR signal size utilizing numerous alanine dosimeters for 10 MV X-rays generated from a therapeutic LINAC to determine its feasibility for use as a quality control measure for radiotherapy equipment. The overall flowchart of the experiments conducted in this study is shown in Figure 4. In summary, the alanine dosimeter was irradiated with 10-MV X-rays from the LINAC, and the resulting signal was measured using an EPR spectrometer. Based on the obtained EPR signal data, the signal intensity was analyzed as a function of absorbed dose and number of alanine dosimeters. Dose–response curves were constructed for each condition, and their measurement uncertainties were evaluated.

### 4.1. Reference Irradiation of Alanine Dosimeter Radiation

**Figure 5:**
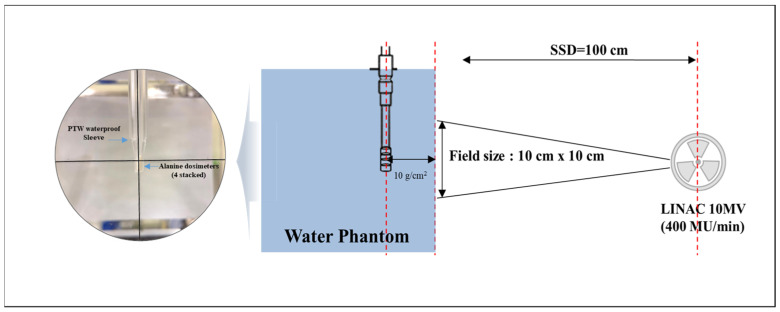
Standard irradiation conditions for alanine dosimetry at LINAC.

In this study, reference irradiation of alanine dosimeters was performed using the LINAC (Elekta Infinity, Elekta, Stockholm, Sweden) installed at the Dongnam Institute of Radiological & Medical Sciences. This institute is a globally recognized calibration laboratory (accreditation no. KC14-297) and an accredited calibration laboratory for ion chamber dosimeters. The LINAC 10 MV photon dosimetry was performed using an ionization chamber dosimeter (TM30013, PTW, Freiburg, Germany; Serial No. 6376; calibration factor: N_Dw_ = 5.432; certificate no.: 2017-R-167) and an ionizing ammeter (PTW Unidose Webline, PTW, Freiburg, Germany). The measurement conditions followed the guidelines outlined by the American Association of Physicists in Medicine (AAPM) TG-51 [17] and IAEA TRS-398 [18]. A water phantom measuring 30 × 30 cm^2^ with a source-to-surface distance (SSD) of 100 cm and water depth of 10 cm was utilized, with dose rates at 10-MV (400 MU/min) energy being measured. The irradiation conditions for constructing the dose-response curves of the alanine dosimeter in this study are shown in Figure 5 and Table 5. During standard irradiation, four alanine dosimeters were vertically stacked, sealed with Parafilm for waterproofing, and placed inside a PTW waterproof sleeve (T41023.1.160, PTW, Freiburg, Germany; polyethylene-based). The sleeve is composed of thin low atomic number plastic material, and its attenuation effect under megavoltage photon beam conditions is considered negligible.

### 4.2. EPR Spectrometer and Alanine Dosimeter

Alanine, with the molecular structure of CH_3_(NH_2_)CHCOOH, is an amino acid known to generate stable free radicals upon exposure to radiation. The quantity of free radicals generated is directly proportional to the amount of radiation absorbed. The alanine dosimeter utilized in this study (E2044562, Bruker BioSpin GmbH, Rheinstetten, Germany) was bound with L-α-alanine (80%) and polyethylene (20%), measured 5 mm in diameter, 3 mm in height, and had a mass of (64.5 ± 0.5) mg.

The Dongnam Institute of Radiological & Medical Sciences is operated as a KOLAS testing institute, adhering to the Standard designation of “Practice for use of the alanine-EPR dosimetry system” according to the KS Q ISO/IEC 17025:2017 standards [19]. All experiments were conducted using the same dosimeter with Batch No. T020604. EPR/alanine dosimetry was performed according to the recommendations of ASTM 51607 [20].

ASTM 51607 specifies the absorbed dose measurement range of an alanine dosimeter as 1 Gy–150 kGy. Wieser et al. validated that the radiation sensitivity of an alanine dosimeter remains constant within the radiation therapy dose range of 0.5 Gy–1 kGy [21]. Consequently, the minimum dose for the alanine dosimeter in this study was established at 1 Gy.

An EPR spectrometer leverages the unique magnetic properties of free radicals for detection and quantification. By placing a reference irradiated alanine dosimeter in the EPR device, the free radicals in the sample absorb microwaves of a specific frequency. The intensity of the absorbed microwaves is directly proportional to the amount of free radicals present in the sample, which correlates to the amount of radiation received. This absorbed dose of radiation can be accurately measured through a dose-response curve. The EPR spectrometer utilized in this study (Bruker E500 ELEXSYS, Bruker BioSpin GmbH, Rheinstetten, Germany) operated in the X-band microwave frequency range (9–10 GHz) for alanine dosimetry measurements. The specific measurement parameters included a microwave frequency of 9.81 GHz, microwave power/ms of 0.6325 mW, modulation amplitudes/ms of 1, 7 G, g-factor at 2.0000, receiver gain of 50 dB, conversion time of 30.16 ms, time constant of 163.84 ms, and number of scans of 10. For measurements involving multiple alanine dosimeters, the pellets were vertically stacked within the quartz sample tube and positioned at the center of the resonator cavity. The same sample holder and insertion depth were maintained for all measurements to minimize positional sensitivity variations within the cavity.

Environmental conditions during storage, irradiation, and readout were controlled to minimize potential fading and signal instability. All alanine dosimeters were stored at room temperature (23 ± 2 °C) with relative humidity below 50%. Irradiation and EPR measurements were conducted under stable laboratory conditions.

The time interval between irradiation and EPR readout was maintained within 24 h for all measurements. According to ASTM 51607 and established alanine dosimetry literature, fading effects within this time frame in the radiotherapy dose range are negligible. Therefore, no additional correction factors for environmental conditions or fading were applied.

### 4.3. Comparison of Dose-Response Curves Based on the Mass of Alanines

**Figure 6:**
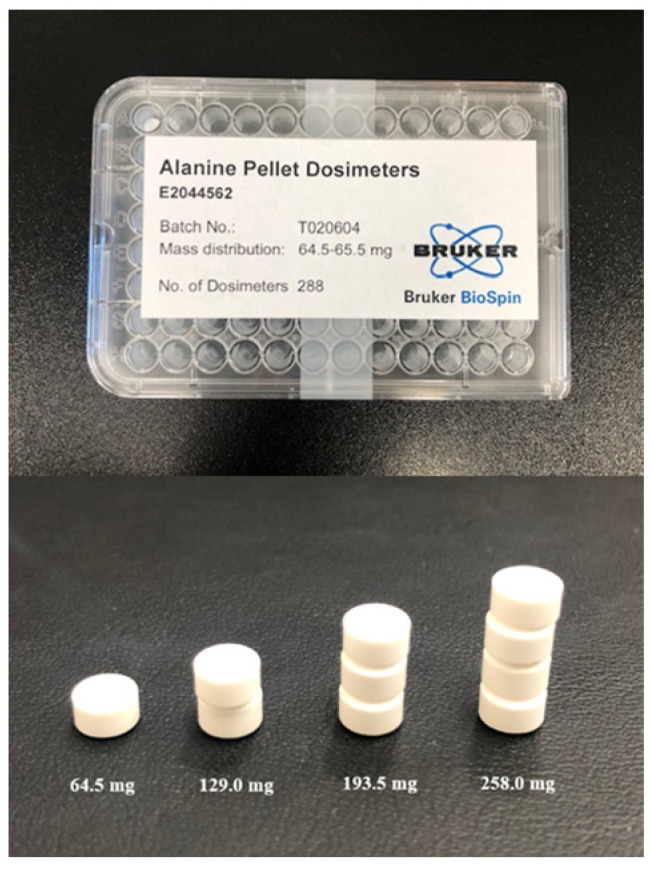
Alanine dosimeter.

A comparison of EPR signals by dose and number was performed by measuring the EPR signals from one, two, three, and four alanine dosimeters at five dose intervals of 1, 5, 10, 15, and 20 Gy. The alanine dosimeter utilized for these measurements is shown in Figure 6. The resulting dose-response curve was constructed as a first-order function by fitting the irradiated dose and the read EPR signal to an interpolation function.

As the dose increased from 1 Gy to 20 Gy, the EPR signal intensified proportionally. In such instances, the calculation adhered to the signal-to-dose response curve, and the dose-dependent parameter of the alanine dosimeter spectrum was evaluated as the vertical peak-to-peak intensity of the dominant peak. The line was fitted using the following linear equation:

*I* = *b*_0_ + *b*_1_*D* (2)

where *I* represents the EPR signal intensity, *D* represents the applied dose, *b*_0_ represents the intercept, and *b*_1_ represents the slope.

The masses depicted in the figure were 64.5, 129.0, 193.5, and 258.0 mg. The coefficients of determination and slopes of the dose-response curves were compared based on these values. Additionally, eight factors influencing the measurement uncertainty of the dose-response curves were identified, including irradiation, storage, and reading, in accordance with international standards and previous research. Among these factors, repeatability, mass determination, and calibration curve fall under A-type uncertainties, indicating variability based on the individual conducting the measurements. Therefore, these values were obtained experimentally. The remaining uncertainty components—namely reference irradiation, EPR reference correction, system drift, temperature correction, and interspecimen contamination—were classified as B-type uncertainties to account for machine- and system-related contributions. The uncertainty associated with reference irradiation was estimated based on the high-dose dosimetry uncertainty tables provided by the National Institute of Standards and Technology (NIST) [22]. The uncertainty contributions for EPR reference correction, system drift, temperature correction, and interspecimen contamination were adopted from Applications of EPR in Radiation Research [23]. Their relative uncertainty values were evaluated as 2.46%, 0.05%, 0.1%, 0.1%, and 0.1%, respectively. The relative combined uncertainty for these components, together with the other identified uncertainty factors (eight in total), was calculated using the law of propagation of uncertainty. These literature-based values were considered applicable to the present study because the experimental configuration—including X-band operation (~9–10 GHz), the use of a Bruker E500 ELEXSYS spectrometer, alanine pellet geometry, and the radiotherapy dose range—is consistent with the measurement conditions described in the referenced sources.

## 5. Conclusions

In this study, we assessed the effectiveness of EPR/alanine dosimetry systems in ensuring the quality control of medical linear accelerators. The results indicated that the dose-response curves of EPR/alanine dosimeters were consistently reliable. Alanine dosimeters, in particular, demonstrated a distinct advantage in accurately assessing exposure doses. The results confirmed that EPR/alanine dosimetry systems could be confidently utilized as measurement tools for the quality control of medical linear accelerators.

Furthermore, the sensitivity and precision of the EPR signal and dose-response curve increased upon increasing the number of alanine dosimeters. Notably, the most reliable results were obtained when four alanine dosimeters were simultaneously measured. However, given that the spatial distribution pattern of the radiation is a crucial aspect of the quality control of medical linear accelerators, increasing the number of alanine dosimeters is expected to be disadvantageous in terms of spatial resolution. Although increasing the number of alanine dosimeters results in proportional enhancement of the detected EPR signal, practical limitations must be considered. The total measurable mass may be constrained by the irradiation field size, beam uniformity, and dose distribution within the measurement volume. In addition, the effective resonance cavity volume of the EPR spectrometer imposes physical limits on the maximum sample volume that can be accurately measured. Therefore, while signal sensitivity improves with increasing mass, optimization requires balancing signal enhancement with spatial resolution and instrumental constraints. Specifically, the 12 mm effective length of a four-pellet stack may limit its application in measuring steep dose gradients, such as penumbra regions, due to volume averaging effects. Therefore, further investigations are required to determine the optimal number of alanine dosimeters based on the specific spatial requirements of each quality control task.

## Figures and Tables

**Table 1:** Repeatability uncertainty.

No	64.5 mg	129.0 mg	193.5 mg	258.0 mg
1	0.2434	0.5449	0.8545	1.1426
2	0.2424	0.5440	0.8506	1.1402
3	0.2420	0.5449	0.8569	1.1429
4	0.2417	0.5498	0.8653	1.1447
5	0.2418	0.5530	0.8610	1.1463
6	0.2447	0.5521	0.8592	1.1468
7	0.2411	0.5545	0.8641	1.1462
8	0.2401	0.5417	0.8564	1.1392
9	0.2405	0.5460	0.8507	1.1392
10	0.2407	0.5427	0.8557	1.1402
Average	0.2418	0.5474	0.8574	0.0030
Standard deviation	0.0014	0.0046	0.0050	0.0030
Uncertainty (%)	0.58	0.84	0.59	0.27

**Table 2:** Uncertainty of mass determination.

Parameter	64.5 mg	129.0 mg	193.5 mg	258.0 mg
Uncertainty of Scale	0.3	0.3	0.3	0.3
Resolution of Scale	0.06	0.06	0.06	0.06
Mass Average (mg)	64.8	129.6	194.3	259.0
Standard Deviation of Mass	0.00	0.00	0.04	0.00
Uncertainty (%)	0.47	0.24	0.16	0.12

**Table 3:** Uncertainty of the calibration curve.

Parameter	64.5 mg (%)	129.0 mg (%)	193.5 mg (%)	258.0 mg (%)
1 Gy	−0.50	5.41	3.65	0.06
5 Gy	−0.38	0.22	−0.06	−0.14
10 Gy	0.95	−1.59	0.25	0.02
15 Gy	−0.24	0.55	−1.00	−0.04
20 Gy	0.16	0.00	0.59	−0.04
Uncertainty	0.68	3.27	2.22	0.10

**Table 4:** Summary of the uncertainty budget for the EPR/alanine dosimetry system (k = 1).

Uncertainty Component	Type (A/B)	Relative Uncertainty (%)
Repeatability	A	0.58–0.84
Mass determination	A	0.12–0.47
Calibration curve	A	0.10–3.27
Reference irradiation	B	2.46
EPR reference correction	B	0.05
System drift	B	0.10
Temperature correction	B	0.10
Interspecimen contamination	B	0.10
Combined uncertainty (k = 1)		2.49–4.19

**Table 5:** Irradiation parameters.

Parameter	Value
Energy	10 MV
SSD	100 cm
Depth	10 g/cm^2^ in water
Field size	Square 10 × 10 cm^2^ in water phantom surface

## Data Availability

The data presented in this study are available within the article.

## Abbreviations

The following abbreviations are used in this manuscript:

EPR	Electron paramagnetic resonance
LINAC	Linear accelerator
SSD	Source-to-surface distance

## References

[1] Tai D.T., Loan T.T.H., Sulieman A., Tamam N., Omer H., Bradley D.A. (2021). Measurement of neutron dose equivalent within and outside of a linac treatment vault using a neutron survey meter. Quantum Beam Sci..

[2] Lee J., Kim W.C., Yoon W.S., Rim C.H. (2021). Implications of radiotherapy utilization in Korea from 2010 to 2019. J. Korean Med. Sci..

[3] Marrale M., Schmitz T., Gallo S., Hampel G., Longo A., Panzeca S., Tranchina L. (2015). Comparison of EPR response of alanine and Gd_2_O_3_-alanine dosimeters exposed to TRIGA Mainz reactor. Appl. Radiat. Isot..

[4] Wieser A., Darroudi F. (2014). EPRBioDose 2013: EPR applications and biological dosimetry. Radiat. Environ. Biophys..

[5] Choi K.H. (2023). Quality Control of Medical Imaging Equipment Using Control Charts: Statistical Aspects and Utilization of SPSS. J. Next-Gener. Converg. Technol. Assoc..

[6] Jeong W.J., Kim D.H., Lee H.Y. (2022). A Study on Image Quality and Dose according to Changes in Tube Voltage and Image Reconstruction Algorithm in Cervical CT Scan. J. Next-Gener. Converg. Technol. Assoc..

[7] Sara E., Bergstrand S. (2000). Calibration of Alanine Dosimeters.

[8] Wielopolski L., Maryanski M., Ciesielski B., Forman A., Reinstein L.E., Meek A.G. (1987). Continuous three-dimensional radiation dosimetry in tissue-equivalent phantoms using electron paramagnetic resonance in L-α-alanine. Med. Phys..

[9] Schaeken B., Cuypers R., Lelie S., Schroeyers W., Schreurs S., Janssens H., Verellen D. (2011). Implementation of alanine/EPR as transfer dosimetry system in a radiotherapy audit programme in Belgium. Radiother. Oncol..

[10] Marrale M., Longo A., Russo G., Casarino C., Candiano G., Gallo S., Carlino A., Brai M. (2015). Dosimetry for electron Intra-Operative RadioTherapy: Comparison of output factors obtained through alanine/EPR pellets, ionization chamber and Monte Carlo-GEANT4 simulations for IORT mobile dedicate accelerator. Nucl. Instrum. Methods Phys. Res. Sect. B.

[11] Wagner D.M., Hüttenrauch P., Anton M., von Voigts-Rhetz P., Zink K., Wolff H.A. (2017). Feasibility study of entrance and exit dose measurements at the contra lateral breast with alanine/electron spin resonance dosimetry in volumetric modulated radiotherapy of breast cancer. Phys. Med. Biol..

[12] Anton M., Büermann L. (2015). Relative response of the alanine dosimeter to medium energy X-rays. Phys. Med. Biol..

[13] Marrale M., Abbene L., d’Errico F., Gallo S., Longo A., Panzeca S., Tana L., Tranchina L., Principato F. (2017). Characterization of the ESR response of alanine dosimeters to low-energy Cu-target X-tube photons. Radiat. Measur..

[14] Anton M. (2006). Uncertainties in alanine/ESR dosimetry at the Physikalisch-Technische Bundesanstalt. Phys. Med. Biol..

[15] National Institute of Standards and Technology (NIST) (2013). Medical (Archive): Small-Field Therapy Dosimetry Studies Using Alanine/EPR. https://www.nist.gov/programs-projects/medical-archive-small-field-therapy-dosimetry-studies-using-alanineepr.

[16] Hjørringgaard J.G., Miller A., Andersen C.E., Cloetta D., Wandfluh W., Tallentire A. (2022). Comparison of the microbicidal effectiveness of 150 kV X-rays and cobalt-60 gamma rays. Radiat. Phys. Chem..

[17] Almond P.R., Biggs P.J., Coursey B.M., Hanson W.F., Huq M.S., Nath R., Rogers D.W. (1999). AAPM’s TG-51 protocol for clinical reference dosimetry of high-energy photon and electron beams. Med. Phys..

[18] International Atomic Energy Agency (2000). IAEA TRS 398: Absorbed Dose Determination in External Beam Radiotherapy: An International Code of Practice for Dosimetry Based on Standards of Absorbed Dose to Water.

[19] (2017). General Requirements for the Competence of Testing and Calibration Laboratories.

[20] (2022). Standard Practice for Use of an Alanine-EPR Dosimetry System.

[21] Wieser A., Lettau C., Fill U., Regulla D.F. (1993). The influence of non-radiation induced ESR background signal from paraffin-alanine probes for dosimetry in the radiotherapy dose range. Appl. Radiat. Isot..

[22] National Institute of Standards and Technology (NIST) (2013). Basic Metrology: High-Dose Dosimetry Uncertainty Tables. https://www.nist.gov/programs-projects/basic-metrology-high-dose-dosimetry-uncertainty-tables.

[23] Lund A., Shiotani M. (2014). Applications of EPR in Radiation Research.

